# Multiple incomplete surgical and procedural interventions correlate with hyperprogression of a pleomorphic dermal sarcoma – a case report

**DOI:** 10.1093/jscr/rjag399

**Published:** 2026-05-27

**Authors:** Sin Yee (Amelie) Lim, Hy B Dao, Fritz C Eilber, Joseph G Crompton, Tyler R McCaw

**Affiliations:** David Geffen School of Medicine, University of California Los Angeles, Los Angeles, CA 90095, United States; Department of Surgery, Division of Surgical Oncology, University of California Los Angeles, Los Angeles, CA 90095, United States; Department of Surgery, Division of Surgical Oncology, University of California Los Angeles, Los Angeles, CA 90095, United States; Jonsson Comprehensive Cancer Center, Sarcoma Oncology Program, University of California Los Angeles, Los Angeles, CA 90095, United States; Department of Surgery, Division of Surgical Oncology, University of California Los Angeles, Los Angeles, CA 90095, United States; Jonsson Comprehensive Cancer Center, Sarcoma Oncology Program, University of California Los Angeles, Los Angeles, CA 90095, United States; Department of Surgery, Division of Surgical Oncology, University of California Los Angeles, Los Angeles, CA 90095, United States; Broad Center of Regenerative Medicine and Stem Cell Research, University of California Los Angeles, Los Angeles, CA 90095, United States

**Keywords:** pleomorphic dermal sarcoma, surgical trauma, residual disease, incomplete oncologic surgery, tumor hyperprogression

## Abstract

Complete surgical resection is the cornerstone of curative-intent treatment for solid malignancies. However, residual disease—whether microscopic or gross—remains in a substantial proportion of cases. Emerging preclinical and clinical data suggest that surgical or procedural trauma in the presence of residual malignancy may be associated with accelerated tumour progression, yet this phenomenon remains poorly characterized in humans. We report the case of a 70-year-old woman with pleomorphic dermal sarcoma who underwent multiple surgical and interventional procedures without complete tumour clearance. Following interventions in which gross residual disease remained *in situ*, the patient experienced rapid local tumor regrowth and fulminant metastases. Quantitative assessment of tumour growth kinetics demonstrated marked acceleration following procedures that left macroscopic disease, consistent with hyperprogression. This case adds to a growing body of evidence suggesting that surgical or procedural manipulation in the presence of gross residual malignancy may be associated with accelerated disease progression.

## Introduction

The goal of curative-intent oncologic surgery is to remove the entirety of a patient’s cancer; however, this is not always feasible. Among the 10 most common solid cancers in the United States, positive surgical margins occur in 5%–35% of surgeries. Often, this represents microscopic disease, or minimal residual disease, and is found on pathologic analysis. In other cases, grossly visible disease can be left. This led us to question: what happens to residual cancer following surgical and/or procedural trauma?

Because inflammation can influence tumour proliferation, invasion, and angiogenesis, it is biologically plausible that procedural trauma in the setting of residual malignancy may promote inflammation and thereby tumour acceleration. Animal models have demonstrated that surgical trauma can accelerate progression of residual disease, typically measured by development of metastases [1]. Emerging clinical data in colorectal cancer, ovarian cancer, and pleomorphic liposarcoma have also suggested acceleration of residual disease following an initial incomplete oncologic surgery, which we term ‘surgically-induced hyperprogression’ [2–4]. A similar phenomenon across multiple cancer histologies suggests this observation may be driven by conserved pathophysiologic mechanisms. It follows that surgical or procedural trauma may activate inflammatory pathways, which can also participate in progression and aggressiveness of residual cancer.

Herein, we present a case of a patient with pleomorphic dermal sarcoma who underwent repeated surgical and procedural interventions and then experienced rapid hyperprogression of both local and systemic (metastatic) disease.

## Case report

The patient is a 70-year-old female with a remote history of tonsilar squamous cell carcinoma metastasic to the left neck treated with partial pharyngectomy and left modified radical neck dissection. She received adjuvant radiotherapy to the resection bed. Afterwards, the patient never had evidence of recurrence.

**Figure 1:**
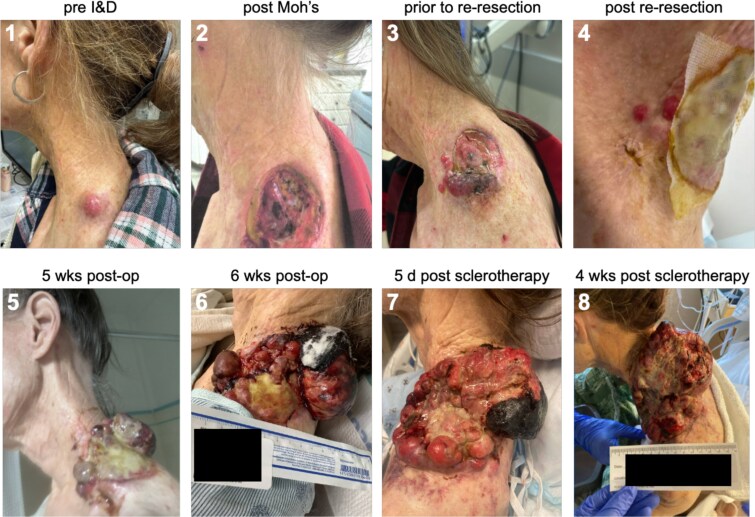
Clinical progression of the patient’s left neck pleomorphic dermal sarcoma at different stages in her treatment course. From left-to-right, top-to-bottom, images are numbered from 1 to 8 according to stage of treatment relative to different surgical and procedural interventions.

More recently, the patient presented to the emergency department following development of a new left neck mass over several months. On physical exam, the mass was 2.5 cm in diameter and mobile (Fig. 1). Computed tomography (CT) neck with contrast demonstrated a 2.4 cm vascular enhancing mass, which was clinically suspected to represent an infected cyst and was managed with incision and drainage (I&D); however, there was no return of purulence. At that time, she was referred for surgical consult and biopsy.

Three weeks later, she underwent Moh’s micrographic surgery. At that time the lesion was 5.2 × 4.8 cm and was removed in a single stage. Initial pathology revealed a de-differentiated malignancy, consistent with an atypical fibroxanthoma. Subsequent outside pathology review suggested the lesion to be a high-grade spindled and epithelioid malignancy.

**Figure 2:**
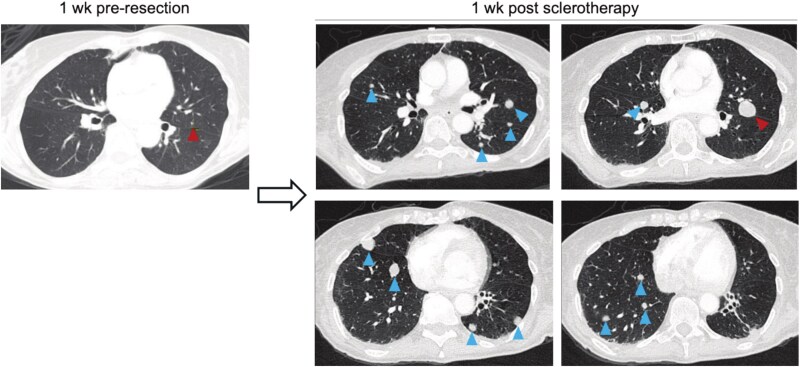
Development of metastatic disease throughout patient’s treatment course. CT chest with contrast is depicted after Moh’s and prior to radical left neck resection (left) and follow-up imaging 1 week after sclerotherapy. Red arrow marks the initial metastatic lesion. Blue arrows indicate subsequent metastases that developed following surgical/procedural interventions.

Five weeks later, she was evaluated by surgical oncology. A staging CT demonstrated a heterogeneous enhancing exophytic mass in left neck measuring 4.7 × 5.4 × 2.0 cm and a single 3.5 mm indeterminant lesion (possible metastasis) in the left lung (Fig. 2). One week later the patient underwent radical resection of the left neck lesion with en bloc resection of two clinically suspected satellite lesions. Final pathology demonstrated high-grade pleomorphic dermal sarcoma with positive medial and deep margins. One of two satellite lesions was also high-grade pleomorphic dermal sarcoma with positive peripheral and deep margins.

In the postoperative period, the patient was referred to medical oncology but elected to pursue holistic options. Six weeks after the radical resection, residual disease rapidly regrew and became hemorrhagic, requiring emergency evaluation. A contrast-enhanced CT neck showed a heterogeneously enhancing mass in left neck at site of initially resected lesion, measuring 5.6 × 5.4 cm, as well as a second heterogeneously enhancing mass extending from the primary mass, measuring 3.7 × 6.3 cm. Two new sub-centimeter lung nodules were also noted. She underwent sclerotherapy with interventional radiology to control bleeding.

The lesion continued to progress, and the patient was admitted one week later for continued inpatient care. Physical exam demonstrated a large bulky exophytic mass. CT neck, chest, abdomen, pelvis demonstrated left neck mass 7.3 × 13.6 × 7.0 cm mass involving skin, subcutaneous fat, and adjacent paravertebral structures. Imaging demonstrated rapid interval progression of metastatic disease with >20 pulmonary nodules, a 2.2 × 2.0 cm enlarging left upper chest wall lesion, and new 1.0 cm left adrenal nodule suspicious for metastasis. She then underwent palliative radiotherapy and was transitioned to hospice care. Less than three weeks later she underwent repeat embolization for bleeding and died of progressive disease within 2 weeks.

## Discussion

This case describes a patient with high-grade pleomorphic dermal sarcoma who experienced rapid local recurrence (Fig. 1) and widespread metastatic dissemination (Fig. 2) following multiple surgical and interventional procedures in which gross residual disease remained *in situ*.

**Figure 3:**
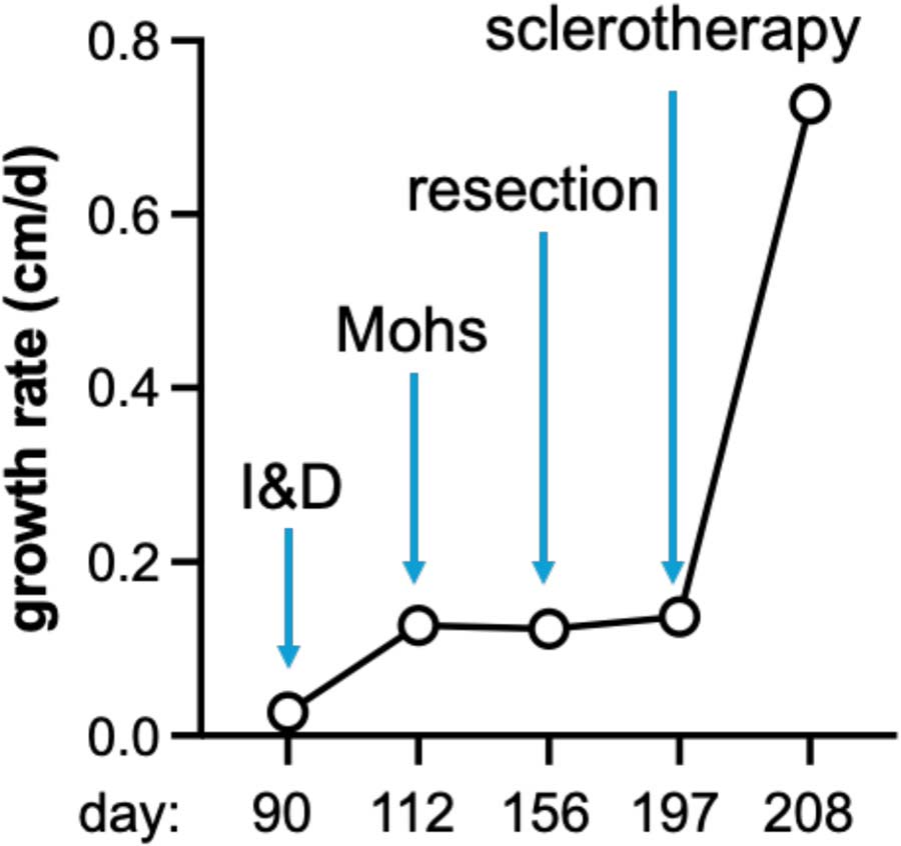
Increasing growth rate of residual disease following each procedural or surgical intervention demonstrates hyperprogression. Growth rate is calculated using difference of greatest tumor diameter between two measurements divided by time between measurements, giving values in cm/day.

To quantify disease progression, we calculated tumour growth rate kinetics throughout the patient’s treatment course (Fig. 3). Strikingly, procedures that removed all but microscopic disease (Moh’s and radical resection; R1 resections) did not appear to alter the residual disease growth rate. In contrast, there was a marked increase in growth rate consistent with hyperprogression of residual disease for procedures that preserved *in situ* tumour (I&D and sclerotherapy). These observations raise the possibility that the presence and volume of residual disease at the time of procedural trauma may influence the magnitude of subsequent tumour acceleration.

As a corollary line of evidence, pre-clinical and clinical data on incomplete tumour ablation provide biological plausibility for this phenomenon. Incompletely ablated tumours in humans and rodent models regrow significantly faster, accompanied by increased inflammatory mediators. Thus, release of inflammatory mediators into circulation is anticipated to accelerate disease progression both locally at the primary tumour site as well as systemically at metastatic sites [5–7].

Importantly, this report does not establish causality. Pleomorphic dermal sarcoma is an aggressive malignancy, and rapid progression may reflect intrinsic tumour biology. Additional contributing factors in this case may include prior radiation exposure, delays in systemic therapy, and selection bias inherent to a single-patient observation. Nevertheless, the temporal relationship between procedural interventions and subsequent acceleration, together with emerging translational data, warrants further investigation.

In conclusion, this case suggests that surgical or procedural manipulation in the presence of gross residual malignancy may be associated with accelerated tumour progression. A deeper understanding of the biological mechanisms underlying this phenomenon may ultimately inform surgical decision-making and therapeutic strategies.

## References

[1] Demicheli R, Retsky MW, Hrushesky WJ et al. The effects of surgery on tumor growth: a century of investigations. Ann Oncol 2008;19:1821–8. 10.1093/annonc/mdn386 18550576

[2] Heaney RM, Shields C, Mulsow J. Outcome following incomplete surgical cytoreduction combined with intraperitoneal chemotherapy for colorectal peritoneal metastases. World J Gastrointest Oncol 2015;7:445–54. 10.4251/wjgo.v7.i12.445 26688707 PMC4678391

[3] Bacalbasa N, Balescu I, Dima S et al. Initial incomplete surgery modifies prognosis in advanced ovarian cancer regardless of subsequent management. Anticancer Res 2015;35:2315–20. 25862895

[4] Fine SA, Lim SY, Siena NM et al. Incomplete cancer surgery correlates with loss of immune surveillance and hyper-progression of disease. J Surg Oncol 2025;132:548–55. 10.1002/jso.70023 40631605

[5] Shi L, Wang J, Ding N et al. Inflammation induced by incomplete radiofrequency ablation accelerates tumor progression and hinders PD-1 immunotherapy. Nat Commun 2019;10:5421. 10.1038/s41467-019-13204-3 31780645 PMC6883042

[6] Markezana A, Goldberg SN, Kumar G et al. Incomplete thermal ablation of tumors promotes increased tumorigenesis. Int J Hyperthermia 2021;38:263–72. 10.1080/02656736.2021.1887942 33612046

[7] Kosari K, Ritchie R, Hunter D et al. Partial radiofrequency ablation causes increased local and remote hepatocellular cancer growth in a rat model. J Am Coll Surg 2003;197:S19.

